# On-Surface Thermal Stability of a Graphenic Structure Incorporating a Tropone Moiety

**DOI:** 10.3390/nano12030488

**Published:** 2022-01-29

**Authors:** Irene R. Márquez, Nerea Ruíz del Árbol, José I. Urgel, Federico Villalobos, Roman Fasel, María F. López, Juan M. Cuerva, José A. Martín-Gago, Araceli G. Campaña, Carlos Sánchez-Sánchez

**Affiliations:** 1 Departamento Química Orgánica, Universidad de Granada (UGR), Unidad de Excelencia de Química UEQ, C. U. Fuentenueva, 18071 Granada, Spain; irenerm@ugr.es (I.R.M.); federicovr@ugr.es (F.V.); jmcuerva@ugr.es (J.M.C.); 2 ESISNA Group, Instituto de Ciencia de Materiales de Madrid (ICMM-CSIC), Sor Juana Inés de la Cruz 3, 28049 Madrid, Spain; n.ruizdelarbol@gmail.com (N.R.d.Á.); mflopez@icmm.csic.es (M.F.L.); gago@icmm.csic.es (J.A.M.-G.); 3 Nanotech@surfaces Group, Empa, Swiss Federal Laboratories for Materials Science and Technology, Überlandstrasse 129, 8600 Dübendorf, Switzerland; jose-ignacio.urgel@imdea.org (J.I.U.); roman.fasel@empa.ch (R.F.); 4 Department of Chemistry, Biochemistry and Pharmaceutical Sciences, University of Bern, Freiestrasse 3, 3012 Bern, Switzerland

**Keywords:** on-surface synthesis, graphene nanoribbons, retro-Buchner reaction, intramolecular rearrangement, scanning tunneling microscope, non-contact atomic force microscope, CO functionalized tip, X-ray photoelectron spectroscopy

## Abstract

On-surface synthesis, complementary to wet chemistry, has been demonstrated to be a valid approach for the synthesis of tailored graphenic nanostructures with atomic precision. Among the different existing strategies used to tune the optoelectronic and magnetic properties of these nanostructures, the introduction of non-hexagonal rings inducing out-of-plane distortions is a promising pathway that has been scarcely explored on surfaces. Here, we demonstrate that non-hexagonal rings, in the form of tropone (cycloheptatrienone) moieties, are thermally transformed into phenyl or cyclopentadienone moieties upon an unprecedented surface-mediated retro–Buchner-type reaction involving a decarbonylation or an intramolecular rearrangement of the CO unit, respectively.

## 1. Introduction

Graphene, and its lower-dimensional derivatives, such as graphene nanoribbons or nanographenes, are interesting fundamental and technological materials thanks to their intriguing optoelectronic and magnetic properties [1,2,3]. These properties depend on their structures, making achieving atomic precision during their synthesis essential. So far, this accuracy can solely be obtained through bottom-up approaches, either under solutions or on surfaces under ultra-high vacuum (UHV) conditions—the latter being known as on-surface synthesis (OSS). The former—although very powerful and versatile from a synthetic point of view—fails when large nanostructures are aimed, due to their lower solubility and higher reactivity [4]. On the other hand, OSS allows for the circumvention of these challenges thanks to the highly inert UHV environment, the 2D confinement, and the special catalytic properties of metallic surfaces, while additionally affording a full set of powerful characterization techniques which even allow for the observation of the intramolecular structure of such nanomaterials [5,6]. To date, a wide variety of low-dimensional graphene-based nanostructures have been synthesized through different on-surface chemical reactions [7,8]. It is worth mentioning that, for example, in the blooming field of graphene nanoribbons (GNRs), most of the strategies used to tune their properties have focused on the modification of structural parameters, such as the size, edge structure, chemical composition, or geometry [9]. Other routes based on the alteration of the local aromaticity by the introduction of non-hexagonal rings are starting to be explored [10,11,12,13,14], mainly focusing on lower-order rings, while larger ones, such as seven- or eight-membered rings are typically limited to their combination with lower pentagonal or square rings, respectively [15,16,17,18,19]. Although the introduction of seven- and eight-membered rings in nanographenes has already been demonstrated using a bottom-up approach in solutions and leading to a saddle-shaped distortion [20,21,22,23,24,25,26,27,28], their inclusion as sole defects into on-surface synthesized extended nanostructures remains a challenge. Therefore, if we aim to tune the properties of on-surface-synthesized graphene-based nanostructures through the introduction of higher-order rings, understanding their thermal stability on metallic surfaces becomes crucial.

**Scheme 1:**
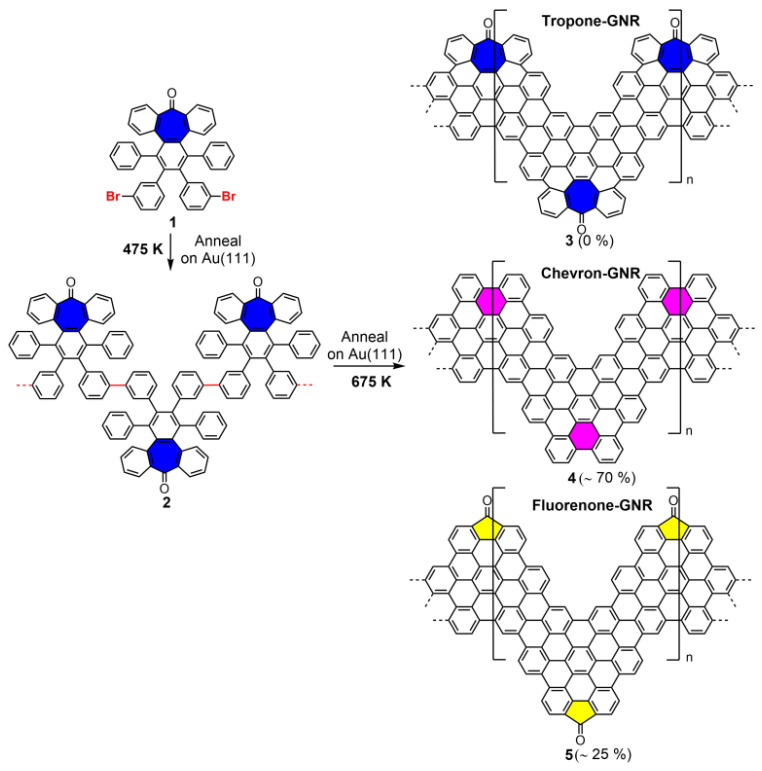
Schematic representation of the different reaction pathways toward the formation of tropone-GNR (**3**), fluorenone-GNR (**5**), and chevron-GNRs (**4**).

Here, we demonstrate that cycloheptatrienone moieties incorporated into the polyphenylene precursor **1** (Scheme 1, ring in blue), designed to yield an edge-distorted heptagon-containing chevron-GNR (**3**), are not stable above 525 K on an Au(111) surface, but reconstruct into phenyl (**4**) or fluorenone-based units (**5**) upon an unprecedented retro-Buchner-type surface-mediated reaction involving a decarbonylation or intramolecular rearrangement, respectively. The resulting GNRs have been characterized through high-resolution scanning tunneling (STM), non-contact atomic force microscopies (nc-AFM), and X-ray photoemission spectroscopy (XPS), and rationalized in terms of analogue solution-based retro-Buchner reactions. This study will contribute to the current understanding of the on-surface thermal stability of isolated higher-order defects in graphenic nanostructures.

## 2. Materials and Methods

### 2.1. Synthetic Details

Unless otherwise stated, all reagents and solvents (CH_2_Cl_2_, hexane) were purchased from commercial sources and used without further purification. Dry toluene was purchased from Scharlau (Barcelona, Spain). Flash column chromatography was carried out using Silica gel 60 (230–400 mesh, VWR, Leuven, Belgium) in the stationary phase. Analytical TLC was performed on aluminum sheets coated with silica gel with the fluorescent indicator UV_254_ (VWR, Leuven, Belgium) and observed under UV light (254 nm) and/or staining with phosphomolybdic acid (5% ethanol solution) and subsequent heating. Preparative TLC was performed on the Silica gel G preparative layer (20 × 20 cm, 1000 microns, Silicycle, Quebec, Canada). All ^1^H and ^13^C NMR spectra were recorded on a Bruker Avance NEO 500 MHz spectrometer (Billerica, MA, USA), at a constant temperature of 298 K. Chemical shifts are reported in ppm and referenced to residual solvents. Coupling constants (*J*) are reported in Hertz (Hz). Standard abbreviations indicating multiplicity were used as follows: m = multiplet, quint. = quintet, q = quartet, t = triplet, d = doublet, s = singlet, b = broad. Assignment of the ^13^C NMR multiplicities was accomplished using DEPT techniques. ESI-TOF mass spectra were recorded using a Waters XEVO GL-XS QT (Buenos Aires, Argentina) of mass spectrometer. IR-ATR spectra were recorded using a Perkin Elmer Spectrum Two IR Spectrometer (Tres Cantos, Spain). Melting points were measured using a Stuart SMP3 (Nemours, France).

### 2.2. Experimental Methods

STM experiments were carried out in three ultra-high vacuum (UHV) systems, two belonging to the ESISNA group (ICMM, Madrid, Spain) and one to the Nanotech@surfaces group (Empa, Zürich, Switzerland). The former ones are equipped with an RT-STM (STM1, Scienta Omicron, Uppsala, Sweden) and an XPS setup (a PHOIBOS 100 1D delay line detector electron/ion analyzer and a monochromatic Al Kα anode (1486.6 eV), Specs Surface Nano Analysis GmbH, Berlin, Germany), respectively, while the latter has an LT-STM/AFM (Scienta Omicron, Uppsala, Sweden). In all systems, the base pressure was below 10^−10^ mbar. STM images shown in this study were acquired at either 77 K or 5 K, as stated in each figure caption. STM images were obtained using the constant current mode, with the bias voltage provided with respect to the sample. Electrochemically etched tungsten tips were used for the measurements, and STM images were analyzed using WSxM software (Madrid, Spain) [29]. Nc-AFM images with CO functionalized tips were acquired at 5 K using the constant height mode. In this case, the tungsten tip, attached to a tuning fork, was functionalized by picking up individual CO molecules from NaCl islands, which were deposited onto the surface after completing the GNR reaction. For these measurements, the sensor was excited to its resonance frequency (22361 Hz) with a constant amplitude of ~80 pm, while the frequency shift was recorded (HF2Li PLL by Zurich Instruments).

XPS measurements were performed using a pass energy of 15 eV. All spectra were calibrated in binding energy (BE) to the Au 4f at 84.0 eV. All fits were carried out using Voigt functions, keeping the Lorentzian full-width half-maximum (FWHM-L) constant during the fitting (0.25, 0.35, and 0.35 eV for C 1s, O 1s, and Br, respectively).

### 2.3. Sample Preparation

Au(111) single crystals (SPL) were prepared using iterative sputtering (Ar^+^, 1 keV) and annealing (750 K) cycles until they were judged to be clean through STM. Compound **1** was deposited onto the Au(111) surface through thermal sublimation at 510 K using a 6-fold ORMA evaporator (Mantis Deposition). The typical deposition rate used was approximately 1 Å/min, as determined by a quartz micro-balance.

## 3. Results and Discussion

Monomer **1**, incorporating both a tropone moiety and aryl bromides, was synthesized in a straightforward manner following a previously described procedure (see details and Figures S1 and S2 in Supplementary Materials) [22]. Scheme 1 shows an illustrative representation of the targeted reaction pathway to incorporate tropone units (blue rings) into well-defined graphenic nanostructures, namely modified chevron-GNRs (tropone-GNR **3**, top part). In principle, it would be expected that when the heptagon-containing polyphenylene monomer **1** is deposited on the Au(111) surface held at 475 K and then post-annealed at 675 K, tropone-containing oligomers **2** and chevron-GNRs incorporating heptagonal rings **3** should be formed upon sequential Ullmann-like coupling and subsequent cyclodehydrogenation reactions, respectively. However, the analysis of 18 different GNRs using in situ nc-AFM images shows that **3** is not obtained and that two other reaction products (**4** and **5**) prevail, as indicated in the reaction scheme. On the one hand, the formation of pristine chevron-GNRs **4** upon the detachment of a CO molecule (surface-mediated decarbonylation) is achieved. On the other hand, we observe the formation of a cyclopentadienone unit (yellow rings) at the external “cape” of the pristine chevron-GNR, in a fluorenone-GNR **5**, with an estimated yield of ~25%. Similar decarbonylation ring transformation upon the loss of atomic/molecular species and intramolecular rearrangements have been previously reported [10,11,30], although never for seven- or higher-membered rings. Interestingly, as discussed later, both pathways can be explained through either a retro-Buchner-type reaction or a decarbonylation process. Additionally, it is worthwhile to note that the remaining 5% is associated with other structures that cannot be clearly identified and which could be related to intramolecular CO rearrangement products or defective reaction products.

**Figure 1:**
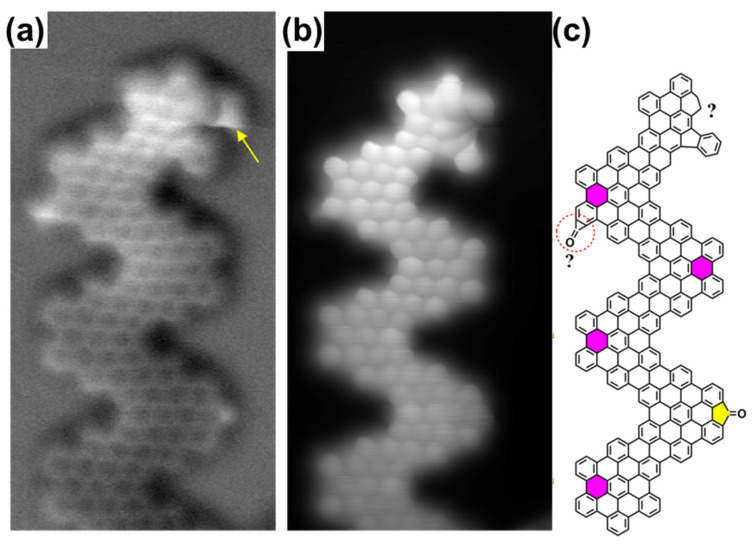
Synthesized chevron-GNRs on Au(111). A constant height (**a**) frequency-shift nc-AFM image and (**b**) a current image simultaneously obtained with a CO-functionalized tip showing the most characteristic variations of the GNRs. Scanning parameters were as follows: (6.5 nm × 3 nm) V = 5 mV, Δz = −2.1 Å @ 0.2 V, and 4 pA. (**c**) A proposed chemical sketch of the GNR structure, including pristine chevron-like (pink) and fluorenone (yellow) segments together with a possible cyclopropenone moiety (red-dashed circle).

Figure 1 summarizes the different structural configurations observed within the same GNR. The first striking conclusion is that although heptagonal rings are preserved during the polymerization step, as judged from the formation of polymeric islands stabilized by O-mediated inter-ribbon H-bonding (vide infra and Section S1 in Supplementary Materials), no tropone moieties survive the final GNR growth process. At some point during the on-surface synthesis, a CO molecule is either detached or rearranged within **2**. In the former case, the new C–C bond formation gives rise to pristine chevron GNRs, as evidenced in the frequency shift and corresponding current images shown in Figure 1a,b and highlighted by the pink 6-membered rings in Figure 1c. In the latter case, a rearrangement of the CO unit and a surface-mediated dehydrogenation reaction, implying the rupture of C–C bonds, results in a fluorenone unit or a cyclopropenone moiety (yellow ring and red-dashed circle in Figure 1c, respectively) linked to one of the external six-membered rings of the pristine chevron-GNR. As shown below, this latter case would be related to an intermediate state in the CO rearrangement sequence. Finally, the upper edge of the GNR presents a distorted geometry, which cannot be determined but must be related to a defective intramolecular cyclodehydrogenation or a defective monomer (yellow arrow in Figure 1a and question mark in Figure 1c), contributing to the 5% undefined structures mentioned above.

**Scheme 2:**
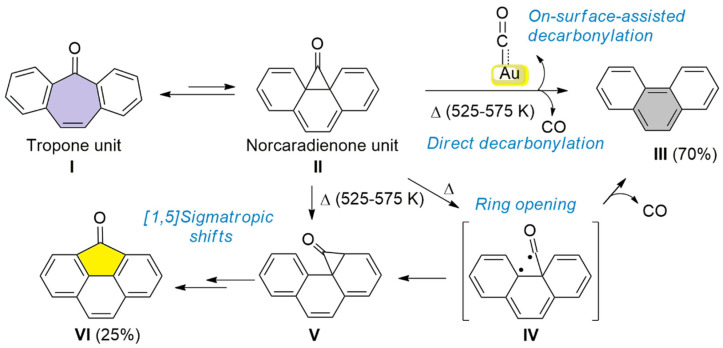
A schematic representation of the possible reaction pathways explaining the conversion of the tropone unit **I** (cycloheptatrienone, in purple) into a cyclopentadienone unit **VI** (yellow) or phenyl **III** (grey) units, including intermediate states.

**Figure 2:**
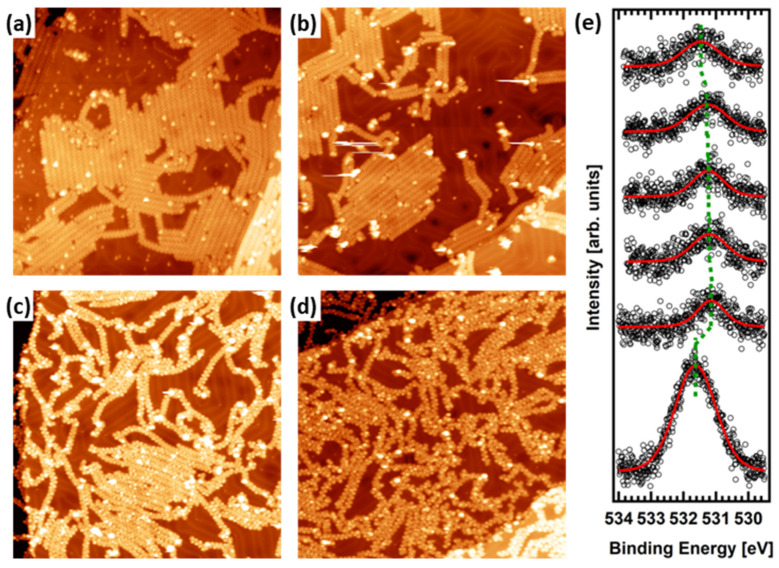
STM images (100 nm × 100 nm) of the thermal evolution of **2** on Au(111) after annealing at (**a**) 475 K, (**b**) 525 K, (**c**) 585 K, and (**d**) 625 K. The STM parameters are as follows: (**a**) I = 30 pA, V = 1.0 V; (**b**) I = 20 pA, V = −1.2 V; (**c**) I = 20 pA, V = −1.0 V; (**d**) I = 20 pA, V = 1.0 V. (**e**) The thermal evolution of the O 1s XPS core-level peak. The green line shows the BE position of the peak maximum. From bottom to top: RT, 435 K, 475 K, 525 K, 575 K, and 625 K, respectively.

In order to elucidate the temperature at which the CO detachment/rearrangement takes place, and to confirm the intact arrival of **1** onto the Au(111) surface upon sublimation, we have carried out a detailed analysis of the STM images showing the polymeric phase (Figure 2), as well as an X-ray photoemission spectroscopy (XPS) study of the O 1s (Figure 2e), C 1s, and Br 3d core-level peaks (Figure S3), as a function of sample temperature. Figure 2a–d present a set of STM images obtained after annealing the surface covered with **1** at 475 K, 525 K, 585 K, and 625 K, respectively. Figure 2a,b show the presence of polymers assembled into islands. It is well-known that oligomeric precursors of pristine chevron-GNRs tend to agglomerate into islands due to an attractive π–π interaction between external interdigitated phenyl rings [31]. However, this stacking disappears upon the planarization of the oligomers into chevron GNRs due to steric repulsion between neighboring in-plane hydrogen atoms [32]. In our case, as the external part of precursor **1** (dibenzoannelated tropone unit, see Scheme 2) is already planarized, interdigitation is unfavorable even at the polymer stage. Still, we observe that the polymers **2** have self-assembled into islands. We attribute this to an attractive hydrogen bonding interaction mediated by the ketone groups in the tropone moieties. For instance, a similar behavior has been reported among N-doped chevron GNRs [32]. If the annealing temperature is increased to 585 K (Figure 2c), some of the oligomers start to detach from the islands. This observation can be rationalized in terms of a partial loss of oxygen atoms from the molecules, indicating that the decarbonylation reaction takes place between 525 K and 585 K, and confirming the intact arrival of precursor **1** onto the surface upon deposition. These results are further supported by the analysis of the XPS core-level peaks. Figure 2e presents the thermal evolution of the O 1s core-level peak (see Section 2 of the ESI for the complete XPS analysis, including C 1s and Br 3d core-level peaks, Figure S3). The bottom curve corresponds to a multilayer deposition of **1** on the clean Au(111) surface held at RT, which has been used as a reference value for the BE of the oxygen in the tropone unit. The obtained BE is characteristic of ketone groups (531.6 eV) [33]. This result confirms that the molecule reaches the surface intact, discards the molecular decomposition already in the crucible, and corroborates the hypothesis of a surface-induced detachment of the CO unit. As we increase the surface temperature to 435 K, the O 1s core-level peak experiences a strong drop in intensity, indicating multilayer desorption. This multilayer desorption is accompanied by an ~ 0.5 eV shift toward a lower BE, which can be attributed either to a charge transfer from the surface toward the molecules or to the increased screening of the signal of the first monolayer [34].

Subsequent annealing steps at 475 K, 525 K, and 575 K do not significantly modify the energy position, suggesting that no important change in the molecular structure of the tropone unit occurs. This range of temperatures corresponds to the formation of the polymer **2** and the initial stages of CDH; therefore, the heptagonal rings seem to remain unaltered upon polymerization by surface-mediated Ullmann coupling. Finally, annealing at 625 K induces a 0.25 eV shift of the peak toward higher BE and an enlargement of the peak width, which could be associated with a small variation in the chemical environment around the ketone group and is compatible with a progressive intramolecular rearrangement of the CO unit, thus giving rise to the fluorenone-GNRs. It is interesting to note that the intensity of the O 1s core-level peak does not significantly change during the whole growth process even though partial decarbonylation takes place. This can be explained by three factors: (i) the XPS signal is quite low as there is only one O atom per molecule, resulting in a low signal-to-noise ratio, as clearly seen in the spectra. This high noise ratio induces a high level of error in the determination of the peak areas, which may account for the low variability in temperature; (ii) the XPS is a macroscopic technique that averages the signal over a rather large area in comparison to STM (mm vs. nm). Additionally, when doing STM, it is common to find areas where the nanostructures are not properly formed; thus, they are contributing to the XPS signal but are not being considered in the STM analysis; (iii) frequency-shift nc-AFM images sometimes reveal the presence of bright dots at the bays of the GNRs. Similar features have been reported for reaction subproducts in the reconstruction of 9-methyl-9H-carbazole into phenanthridine, where they have been attributed to methyl radicals stabilized by the surface [30]. In our case, they could be associated with carbonyl radicals stabilized by the surface and the GNRs, thus also contributing to the O 1s signal. In any case, XPS and STM analysis are in fair agreement (considering that experiments have been carried out in different systems and the temperature has been measured in different ways), thus corroborating that the modification of the tropone moiety must take place between 525 K and 585 K (probably closer to the upper limit of 585 K).

Summing up all the experimental evidence presented, it is proposed that the tropone unit is transformed into a phenyl ring (CO detachment resulting in **4**) or into a cyclopentadienone unit (CO rearrangement resulting in **5**) during the cyclodehydrogenation process (above 525 K). It is interesting to note that similar thermally-induced decarbonylation processes have been reported for fluorenone-based chevron-GNRs on Au(111) [11], although a considerably higher temperature of 625 K and much longer annealing times were needed, thus indicating higher stability of the fluorenone vs. the tropone moieties. This fact rationalizes the transformation of the latter into the former. However, no similar results have been reported for tropone-based GNRs on surfaces. On the other hand, the thermally induced decarbonylation of benzannulated tropones has been previously described in the case of solution chemistry. It implies an electrocyclic ring closure and cheletropic CO extrusion, leading to the corresponding aromatic derivatives during pyrolysis [35,36]. A similar reaction pathway could be suggested in the present case (Scheme 2). In analogy to the well-known cycloheptatriene–norcaradiene equilibrium [37], the cycloheptatrienone unit in **1** (**I**, Scheme 2) would be in equilibrium with the norcaradienone intermediate **II** through electrocyclic ring closure. Subsequently, **II** can decompose directly through a cheletropic extrusion of CO (transition from **II** to **III**) or via diradical **IV**, yielding, in both cases, a phenantrene moiety **III** (resulting in **4**) [35,38]. Several mechanisms could explain the formation of norcaradienone **V**, either by intramolecular rearrangement via diradical **IV** or by a sequence of [1,5] sigmatropic shifts of the cyclopropanone subunit [35,39]. Ring opening/closure from norcaradienone **V** and final dehydrogenation would form the fluorenone unit in **VI** (as in the case of **5**, Scheme 1). Within this context, it is also worth noting that the described pyrolysis of tropones without surface interaction implies higher temperatures than the ones reached in our OSS case [35]. Therefore, we should also take into consideration the role of the gold surface and the presence of Au adatoms in the reaction pathway, which is related to the retro-Buchner reaction mediated by Au(I), as reported by Echavarren et al. [40,41]. In that case, cycloheptatrienes, in equilibrium with norcaradienes, can react with cationic Au(I) complexes to obtain Au(I) carbenes and an aromatic system, which could match with the phenanthrene unit in the pristine GNR generated in our case. In this sense, it should be noted that the adatoms present on the surface at elevated temperatures could play the role of these complexes, as it has been demonstrated that they can play an active role in catalyzing reactions [42,43,44]. Our results can, therefore, be explained by a retro–Buchner reaction that implies a decarbonylation process forming a gold carbonyl. Thus, these results would represent the first example of an on-surface retro–Buchner-type reaction.

## 4. Conclusions

We have shown, by means of STM, nc-AFM, and XPS, an unprecedented conversion of a cycloheptatrienone unit into a phenyl group or a cyclopentadienone unit during the cyclodehydrogenation step through the detachment or rearrangement of the CO unit, respectively. A reaction pathway, based on a gold-mediated CO extrusion reaction, is proposed.

This work aids in the understanding of the stability of higher-order non-hexagonal rings incorporated into graphenic nanostructures as a path towards the tailored engineering of their properties through the introduction of out-of-plane structural distortions, thus expanding the toolbox for the bottom-up synthesis of atomically precise carbon-based nanostructures.

## Data Availability

Not applicable.

## Supplementary Materials

The following are available online at https://www.mdpi.com/article/10.3390/nano12030488/s1, references [45,46,47,48] are cited in the supplementary materials. Figure S1: 1H NMR (500 MHz, CDCl3) spectrum of compound 1, Figure S2: DEPT-135 NMR (126 MHz, CDCl3) (top) and 13C NMR (126 MHz, CDCl3) (bottom) spectra of compound 1, Figure S3: Thermal evolution of the (a) C 1s and (b) Br 3d core level peaks.
