# Supplementary material for: On-Surface Thermal Stability of a Graphenic Structure Incorporating a Tropone Moiety
Source: Nanomaterials (Basel). 2022 Jan 29;12(3):488. doi: 10.3390/nano12030488 (PMC8837919; doi:10.3390/nano12030488)
Supplement: Supplementary file 1 [file nanomaterials-12-00488-s001.zip › nanomaterials-1510478-supplementary.pdf]

nanomaterials

MDPI

Electronic Supplementary Material

On-Surface Thermal Stability of a Graphenic Structure Incorporating a Tropone Moiety

Irene R. Márquez [1], Nerea Ruiz del Árbol [2], José I. Urgel [3], Federico Villalobos [1], Roman Fasel [3,4], María F. López [2], Juan M. Cuerva [1], José A. Martín-Gago [2], Araceli G. Campaña [1,*] and Carlos Sánchez-Sánchez [2,*]

[1] Departamento Química Orgánica, Universidad de Granada (UGR), Unidad de Excelencia de Química UEQ, C. U. Fuentenueva, 18071 Granada, Spain; irenerm.119@gmail.com (I.R.M.); federicovr@ugr.es (F.V.); jmcuerva@ugr.es (J.M.C.)

[2] Instituto de Ciencia de Materiales de Madrid (ICMM-CSIC), Sor Juana Inés de la Cruz 3, 28049 Madrid, Spain; n.ruizdelarbol@gmail.com (N.R.d.Á.); mflopez@icmm.csic.es (M.F.L.); gago@icmm.csic.es (J.A.M.-G.)

[3] Empa, Swiss Federal Laboratories for Materials Science and Technology, Überlandstrasse 129, CH-8600 Dübendorf, Switzerland; jose-ignacio.urgel@imdea.org (J.I.U.); roman.fasel@empa.ch (R.F.)

[4] Department of Chemistry, Biochemistry and Pharmaceutical Sciences, University of Bern, Freiestrasse 3, CH-3012 Bern, Switzerland

* Correspondence: araceligc@ugr.es (A.G.C.); cssanchez@icmm.csic.es (C.S.-S.)

Table of Contents

S1. Synthesis and spectroscopy data of compound 1 2

S2. XPS analysis 4

S3. References 5

S1. Synthesis and spectroscopy data of compound 1

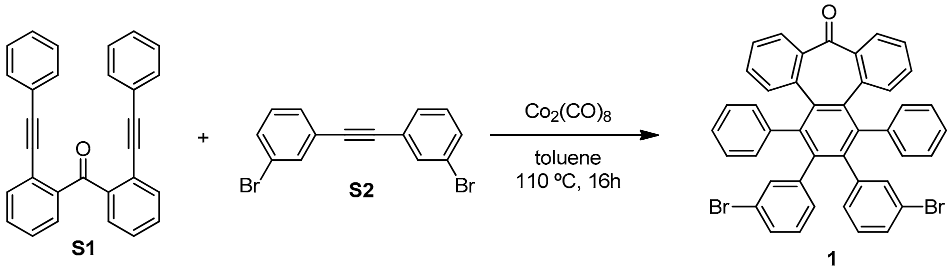

A degassed solution of the dialkyne **S1**[1] (230 mg, 0.60 mmol) in toluene (2 mL) was added to a degassed solution of $Co_2(CO)_8$ (267 mg, 0.78 mmol) in toluene (6 mL), and the mixture was stirred at 110 °C during 30 min. Then, a degassed solution of the 1,2-bis(3-bromophenyl)ethyne **S2**[2] (302 mg, 0.9 mmol) in toluene (2 mL) was added dropwise during 30 min. The reaction was stirred 16 h under argon atmosphere at 110 °C. The mixture was then cooled down to room temperature and the solvent was removed under reduced pressure. The residue was adsorbed on silica gel and purified firstly by column chromatography (SiO_2, Hexane/CH_2Cl_2 : 7/3) and then by preparative TLC (SiO_2, Hexane/CH_2Cl_2 : 7/3) to give the corresponding compound **1** (130 mg, 30%) as a white solid. M.p.: >300°C.

Mixture of two atropisomers (55:45). ^{1}H-NMR (500 MHz, $CDCl_3$): δ = 7.56 (*major isomer*, t, *J* = 1.7 Hz, 1H), 7.50 (*minor isomer*, t, *J* = 1.7 Hz, 1H), 7.41 (dd, *J* = 7.6, 1.1 Hz, 2H), 7.31 – 7.24 (m, 1H), 7.17 – 7.08 (m, 3H), 7.04 (tt, *J* = 7.6, 1.9 Hz, 1H), 7.01 – 6.95 (m, 3H), 6.95 – 6.88 (m, 2H), 6.87 – 6.77 (m, 4H), 6.64 – 6.53 (m, 2H), 6.51 – 6.44 (m, 2H), 6.36 (*minor isomer*, dt, *J* = 7.7, 1.3 Hz, 1H), 6.30 (*major isomer*, dt, *J* = 7.7, 1.3 Hz, 1H). ^{13}C-NMR (126 MHz, $CDCl_3$): δ = 200.2 (C), 200.0 (C), 146.0 (C), 141.87 (C), 141.85 (C), 141.8 (C), 141.6 (C), 141.5 (C), 141.4 (C), 141.3 (C), 140.98 (C), 140.96 (C), 140.93 (C), 140.05 (C), 140.02 (C), 140.0 (C), 135.97 (C), 135.96 (C), 135.95 (C), 134.5 (CH), 134.34 (C), 134.33 (C), 134.32 (C), 134.3(CH), 133.4 (CH), 133.2 (CH), 133.0 (CH), 132.99 (CH), 132.97 (CH), 132.4 (CH), 132.11 (CH), 132.08 (CH), 130.43 (CH), 130.41 (CH), 130.1 (CH), 130.0 (CH), 129.0 (CH), 128.9 (CH), 128.86 (CH), 128.73 (CH), 128.71 (CH), 128.66 (CH), 128.63 (CH), 128.59 (CH), 128.57 (CH), 127.8 (CH), 127.7 (CH), 127.5 (CH), 127.49 (CH), 127.4 (CH), 127.38 (CH), 127.3 (CH), 126.9 (CH), 126.8 (CH), 126.7 (CH), 126.67 (CH), 126.0 (CH), 125.8 (CH), 124.4 (CH), 124.34 (CH), 124.30 (CH), 121.2 (C), 121.1 (C), 120.6 (C), 120.4 (C). IR (ATR): 1684, 1593, 1561, 1292, 1215, 748, 727, 716, 667, 638 cm^{-1}. HR-MS (ESI-TOF): *m/z* calcd. for $C_{43}H_{27}Br_2O[M+H]^+$: 717.0429; found: 717.0419.

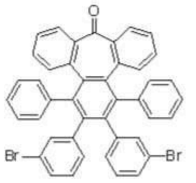

1

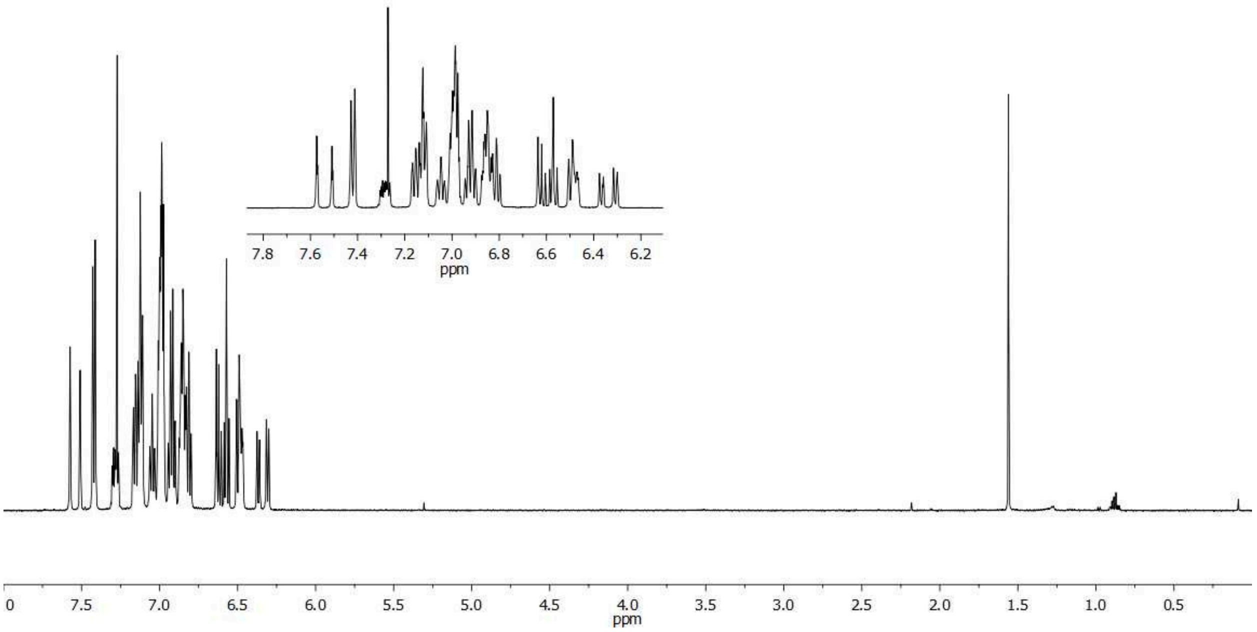

Figure S1. ^{1}H NMR (500 MHz, $CDCl_3$) spectrum of compound **1**.

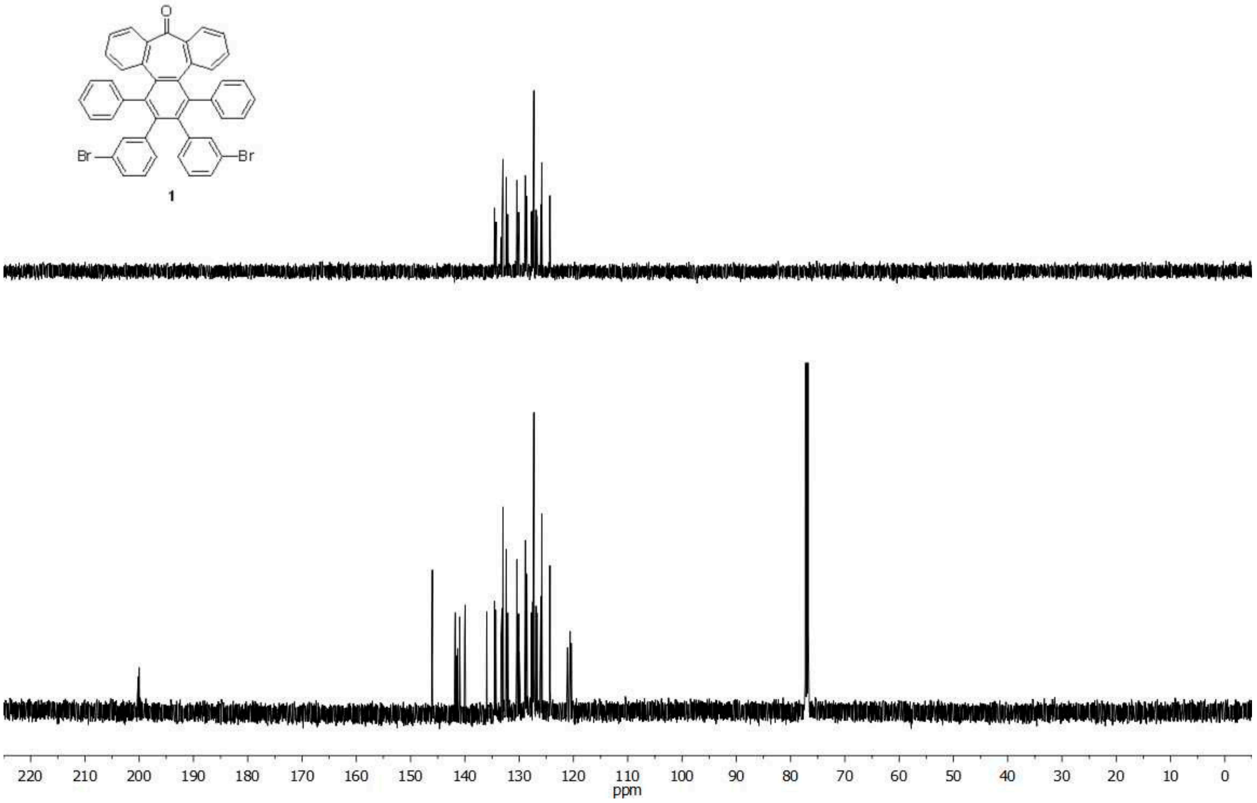

1

Figure S2. DEPT-135 NMR (126 MHz, $CDCl_3$) (top) and ^{13}C NMR (126 MHz, $CDCl_3$) (bottom) spectra of compound **1**.

S2. XPS analysis

Figure S3 shows the thermal evolution of the C 1s and Br 3d XPS core level peaks of **1**, respectively. After the analysis carried out in the main text, further information can be extracted from the C 1s and Br 3d core level peaks. At RT, four components are present in the C 1s peak (Figure S3 a)), associated to the four C species in **1**, namely C–H (blue component), C–C (green component), C-Br (purple component), and C=O (gold component). Interestingly, the area ratios are consistent with molecular stoichiometry. If the system is annealed at 435 K, two clear effects are observed. Firstly, there is an evident decrease in the total area of the C 1s signal, associated to the already mentioned multilayer desorption. Secondly, there is a decrease in the C–Br signal and a concomitant appearance of a small new component at 283.3 eV (cyan curve), which is characteristic of C atoms bound to metal atoms.[3] Consequently, molecular dehalogenation has started at 435 K and some of the so-formed C radicals are stabilized by the Au atoms of the surface. This result is in good agreement with previous similar studies of GNRs on Au(111).[4] As the system temperature is increased up to 625 K, there is a gradual interconversion of C–H signal into C-C one, thus indicating the formation of new C–C bonds upon surface-assisted cyclodehydrogenation, yielding the formation of the GNRs. Interestingly, the C-O component is present at 625 K, thus confirming the presence of fluorenone units in the final GNRs.

If we focus our attention on the thermal evolution of the Br 3d core level peak, we observe the characteristic behavior of molecular precursors giving rise to GNRs on Au(111). At RT, the peak is composed of a single component located at 70.0 eV, as expected for Br atoms attached to the molecule. When the temperature is increased to 435 K, a new component at 67.7 eV appears, associated to Br atoms on the Au surface.[4] This second component increases its area as temperature is ramped from 435 K to 475 K, temperature at which it becomes the sole component, indicating a complete dehalogenation of the molecules. By rising the temperature above 475 K, this component decreases gradually up to its complete disappearance at 575 K as a consequence of Br desorption, in good agreement with reported desorption temperatures.[5]

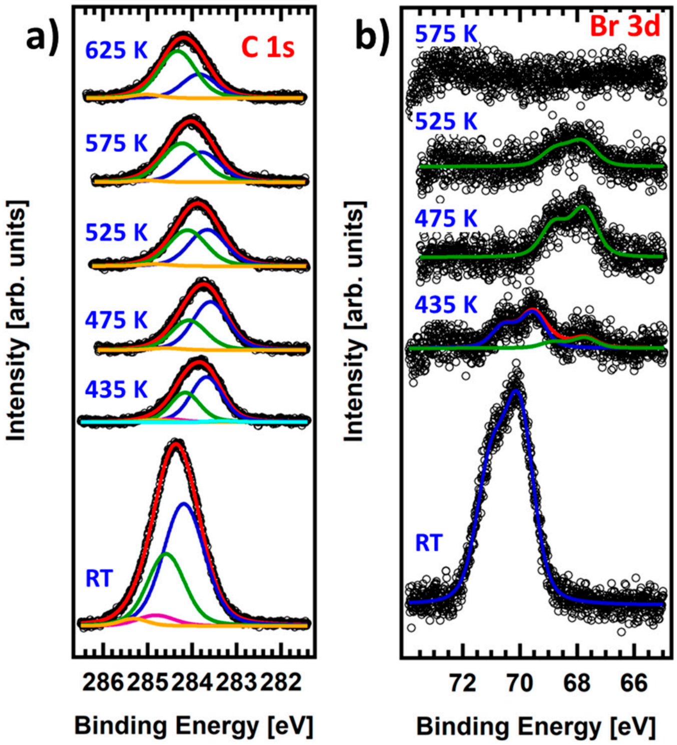

Figure S3. Thermal evolution of the (**a**) C 1s and (**b**) Br 3d core level peaks.

S3. References

1. Márquez, I.R.; Fuentes, N.; Cruz, C.M.; Puente-Muñoz, V.; Sotorrios, L.; Marcos, M.L.; Choquesillo-Lazarte, D.; Biel, B.; Crovetto, L.; Gómez-Bengoa, E.; et al. Versatile Synthesis and Enlargement of Functionalized Distorted Heptagon-Containing Nanographenes. *Chem. Sci.* **2017**, *8*, 1068–1074, doi:10.1039/C6SC02895K.
2. Mio, M.J.; Kopel, L.C.; Braun, J.B.; Gadzikwa, T.L.; Hull, K.L.; Brisbois, R.G.; Markworth, C.J.; Grieco, P.A. One-Pot Synthesis of Symmetrical and Unsymmetrical Bisarylethynes by a Modification of the Sonogashira Coupling Reaction. *Org. Lett.* **2002**, *4*, 3199–3202, doi:10.1021/ol026266n.
3. Simonov, K.A.; Vinogradov, N.A.; Vinogradov, A.S.; Generalov, A.V.; Zagrebina, E.M.; Svirskiy, G.I.; Cafolla, A.A.; Carpy, T.; Cunniffe, J.P.; Taketsugu, T.; et al. From Graphene Nanoribbons on Cu(111) to Nanographene on Cu(110): Critical Role of Substrate Structure in the Bottom-Up Fabrication Strategy. *ACS Nano* **2015**, *9*, 8997–9011, doi:10.1021/acsnano.5b03280.
4. Di Giovannantonio, M.; Deniz, O.; Urgel, J.I.; Widmer, R.; Dienel, T.; Stolz, S.; Sánchez-Sánchez, C.; Muntwiler, M.; Dumslaff, T.; Berger, R.; et al. On-Surface Growth Dynamics of Graphene Nanoribbons: The Role of Halogen Functionalization. *ACS Nano* **2018**, *12*, 74–81, doi:10.1021/acsnano.7b07077.
5. Bronner, C.; Björk, J.; Tegeder, P. Tracking and Removing Br during the On-Surface Synthesis of a Graphene Nanoribbon. *J. Phys. Chem. C* **2015**, *119*, 486–493, doi:10.1021/jp5106218.
